# Tenon's Cyst Presenting as a Long-Term Complication following Incision Cataract Surgery

**DOI:** 10.1155/2013/759267

**Published:** 2013-11-21

**Authors:** Prabhakar Srinivasapuram Krishnacharya

**Affiliations:** Department of Ophthalmology, JSS Hospital, M.G. Road, Mysore, Karnataka 570004, India

## Abstract

*Context.* Tenon's cyst or conjunctival cyst formation is not uncommon late complication of traditional extracapsular cataract surgery; however, few reports are available in the literature. *Aims.* Large cystic swellings were clinically diagnosed as filtering blebs at the cataract incision site in two patients. The purpose of the case presentation is to discuss the factors leading to cyst formation, visual loss and cyst recurrence after its excision. *Patients and Methods. Case 1.* Sixty-one-year-old male patient presented with a bleb at superior limbal region in the right eye, two years after cataract surgery. *Case 2.* A giant bleb was found at the same region in the right eye of a 65-year-old male patient, eight years after cataract surgery. *Results.* Complete excision of the cyst was performed with conjunctival autograft in the first patient and followed up for two years. No recurrence of the cyst was observed. Internal wound gaping was seen on gonioscopy in the second patient. *Conclusions.* Unstable scleral tunnel could explain bleb formation in both the patients. Complete bleb excision with conjunctival auto-graft resulted in closure of the defect with no bleb recurrence during two-year follow-up. Over-filtration causing hypotonic maculopathy was the reason for decreased vision in the second case.

## 1. Introduction

Conjunctival inclusion cyst is a benign condition filled with serous fluid and slimy mucous secretions of congenital or acquired origin. Acquired cysts can occur following strabismus surgery, pars plana vitrectomy, scleral buckling, and Ahmed glaucoma valve insertion [1]. A filtering bleb is an intentionally created passage for aqueous humour into the sub-conjunctival space during glaucoma surgery but might be produced unintentionally following conventional manual small incision cataract surgery. Tendons cyst or conjunctival inclusion cysts that are leaking or not leaking could masquerade as filtration blebs following SICS. The incidence rate from the previous reports on unintended filtering blebs after cataract surgery varied between 1% and 7.7% [2, 3]. Gonioscopic visualization of the fish mouthing of the internal opening was reported by previous authors on unplanned filtering blebs following cataract surgery [4].

Over-filtration of aqueous humour resulted in hypotonic maculopathy and endophthalmitis that leads to loss of vision. Excision of the bleb with closure of the defect would result in improvement of vision even after many years [5].

The present case report highlights the importance of factors producing tenons cyst formation and its effects on vision, and to observe for post excision cyst recurrence after using the conjunctival auto graft for the closure of the defect.

## 2. Patients and Methods

### 2.1. Case 1

**Figure 1:**
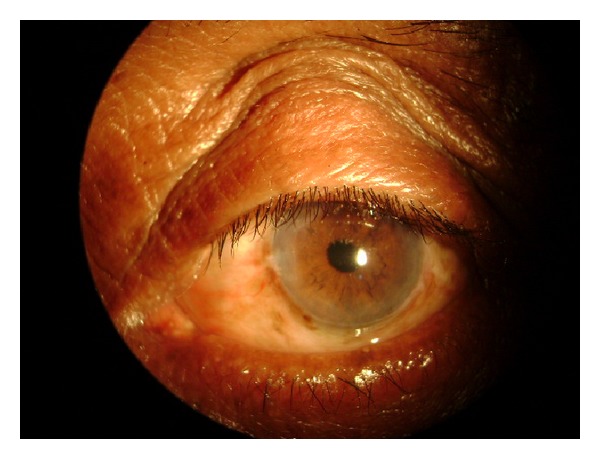
Filtration cystic bleb as seen through the upper eyelid in the first patient.

**Figure 2:**
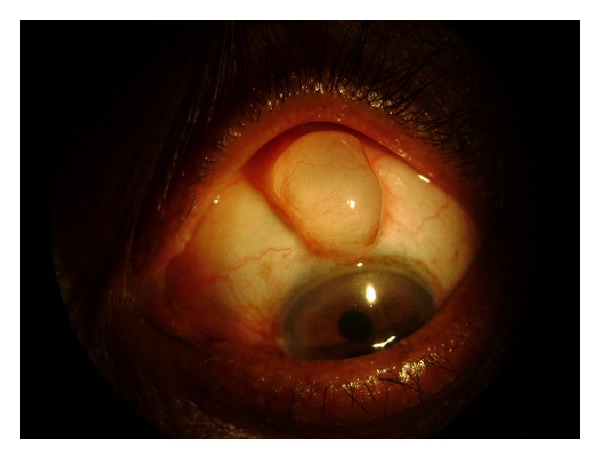
Bleb transillumination as seen in the first case.

A male patient aged about 61 years presented with a swelling in the right eye at the superior limbus for the last six months following cataract surgery with posterior chamber intraocular implantation. There was no evidence of pain or irritating symptoms in the right eye. The swelling was vertically oval measuring 7 mm × 4 mm situated at the scleral tunnel site assuming more or less a spherical shape (Figure 1). Transillumination of the swelling by slit lamp beam revealed a thin, smooth walled cyst containing fluid (Figure 2). The consistency was cystic to firm, nontender with no mobility suggesting that the cyst was adherent to the underlying scleral tissue. The best corrected visual acuity was 20/40 in the right eye and 20/100 in the left eye. The ocular movements and dilated fundus examination were unremarkable. The intraocular pressure was 14 mmHg and 16 mmHg in the right and left eyes, respectively, by rebound tonometry recorded during the morning hours. There was no internal wound gape seen at the internal incision site on gonioscopic examination.

The fluid from the cyst was aspirated under topical 4% lignocaine hydrochloride in the operation theatre. After a week, the cyst refilled again suggesting a communication between the anterior chamber and subconjunctival space necessitating its excision under peribulbar anesthesia. After aspiration of the fluid, there was no shallow or collapse of the anterior chamber. Despite careful dissection, the cyst was ruptured. The supero-temporal conjunctiva was dissected and placed over the large defect and sutured with 8-0 Ethicon silk suture. Vision was stabilized with no recurrence of the cyst observed in two-year follow-up. There were no organisms cultured from the liquid sample that was sent for microbiological examination.

### 2.2. Case 2

**Figure 3:**
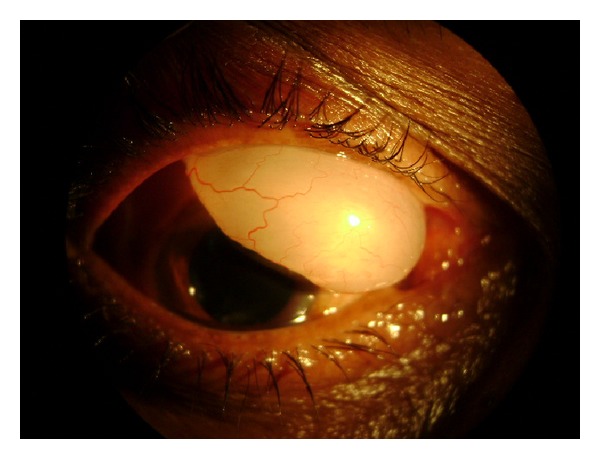
Giant filtration bleb prolapsing through the palpebral aperture in the second patient.

Another male patient aged about 65 years old presented with a giant filtering bleb at the superior limbus in the right eye, eight years after cataract extraction with intraocular implantation. The cyst measured 10 mm × 5 mm prolapsing through the palpebral aperture which had tendency for spontaneous rupture with trivial injury (Figure 3). The best corrected visual acuity was 20/200 and 20/40, respectively, in both the eyes. Ocular movements and dilated fundus examination were unremarkable with normal intraocular pressure. Internal wound gape was seen on gonioscopic visualization of the angle structures. Unfortunately patient refused the surgery.

## 3. Discussion

Manual small incision cataract surgery is the usual technique followed for removal of cataracts in developing countries. Reporting of the incidence of unintended bleb was sparse until the emergence of scleral tunnel evolution. With phacoemulsification, the cyst occurrence is unlikely because of the corneal or limbal side of the incision with minimal disturbance of the conjunctiva. Tenons or conjunctival inclusion cysts are smaller in size, nonprogressive in nature, and transillumination is not fully appreciated when compared to filtration bleb. Because of the elasticity of the conjunctiva filtration blebs are not only large but also progressive in nature and demonstrate considerable internal illumination. The problems arising from the bleb are hypotonic maculopathy causing diminution of vision, post-operative endophthalmitis, and spontaneous rupture.

**Figure 4:**
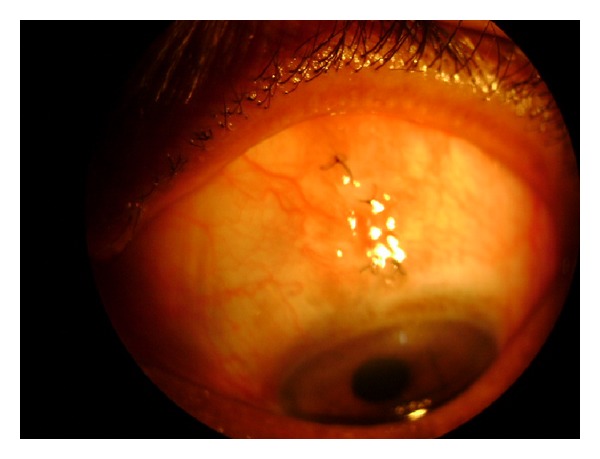
Post-operative appearance of first case after the bleb excision with conjunctival auto graft.

The present first case report described an incompletely encysted bleb as the anterior chamber did not collapse following aspiration of the cyst. Unhealed thin sclera simulating as a spongy filtering tissue might be causing bleb refilling after uncomplicated aspiration. Fish mouthing of the internal opening was observed especially in blebs of shorter duration on gonioscopy. The opening could not be seen on gonioscopic examination in the first case that could be due to the long-standing inflammation. As there was no fish mouthing seen in the first patient and because of the chronic nature of the cyst, we decided to remove the cyst and to close the defect with conjunctival auto-graft to prevent its recurrence (Figure 4). The cyst was completely excised despite the rupture of the cyst during the dissection. In addition to the description of the precautionary aspects of en bloc cyst excision, the author emphasized on maintaining the patience and tolerance during the surgical procedure by the surgeon is most important [6].

The first case was managed by autologous conjunctival grafting that differed from the previous case report on bleb excision, managed by horizontal suturing of the scleral tunnel [4]. The diagnosis and management of internal wound gaping by intra-operative use of gonio prisms were emphasized and very well described by the previous authors [5]. Fortunately, spontaneous resolution of hypotonic maculopathy was observed even after seven years after excision and closure of the defect, as described by the previous reports [7]. Excision of long-standing blebs may be recommended to those patients who have desire for improvement in the vision after the excision of the cyst.

Unanticipated filtering bleb formation after SICS undoubtedly depends on the strength of the scleral tunnel that determines its stability. Therefore the scleral tunnel should be closed by sutures if found unstable. Adequate hydration of the side port normalizes the intraocular pressure which closes the roof and the floor of the tunnel. Other preventive aspects of filtering bleb formation consist of minimum conjunctival dissection and avoiding excessive scleral vessels cauterization that might interfere with the primary wound healing leading to leaky tunnels. It is also important to avoid repeated inclusion of the conjunctiva through the tunnel or carrying the conjunctiva with the intraocular lens while inserting which could implant conjunctival epithelial cells into the anterior chamber.

## 4. Conclusion

In the first case scenario, the vision was preserved in spite of large cystic bleb even after two years compared to the second case that resulted in hypotonic maculopathy leading to profound loss of vision due to over-filtration. Use of conjunctival auto-graft to close the defect after the cyst excision was effective in preserving the vision and preventing the recurrence of the filtering bleb in the first case.
